# Phenotypic plasticity of Nipah virus: An underappreciated driver of spillover risk

**DOI:** 10.1016/j.xinn.2026.101432

**Published:** 2026-05-23

**Authors:** Boyuan Wang, Xueqian Yi, Wei Huang, Xuefang Xu, Chengliang Yin

**Affiliations:** 1 School of Medical Technology and Nursing, Hunan Institute of Traffic Engineering, Hengyang, China; 2 Institute for Intelligent Warning and Rehabilitation Nursing of Major Diseases, Hunan Institute of Traffic Engineering, Hengyang, China; 3 Center for Medical AI Technology Innovation, Zhuhai Fudan Innovation Research Institute, Zhuhai, China; 4 Faculty of Humanities and Arts, Macau University of Science and Technology, Macau, China; 5 Respiratory Disease AI Laboratory in Epidemic Intelligence and Applications of Medical Big Data Instruments, Macau University of Science and Technology, Macau, China; 6 National Institute for Communicable Diseases Control and Prevention, Chinese Center for Disease Control and Prevention, Beijing, China; 7 National Key Laboratory of Intelligent Tracking and Forecasting for Infectious Diseases, Chinese Center for Disease Control and Prevention, Beijing, China; 8 Department of Biomedical Engineering, Faculty of Engineering, Universiti Malaya, Kuala Lumpur, Malaysia

## Phenotypic plasticity as an underrecognized feature of Nipah virus

Since its identification in Malaysia in 1998, Nipah virus (NiV) has been recognized as a priority zoonotic pathogen by the World Health Organization. Most discussions on NiV have focused on its high case fatality rate, which is commonly reported to range from 40% to 75%.^1^ Consequently, NiV is primarily regarded as a highly lethal virus, while its marked variability in clinical manifestations and transmission patterns is frequently neglected.^1^

Unlike the influenza or dengue viruses, which typically display relatively stable symptom profiles, NiV exhibits substantial heterogeneity across outbreaks. During the initial outbreak in Malaysia, infection was predominantly neurotropic, with limited human-to-human transmission associated with pig-related spillover. Conversely, outbreaks in Bangladesh and India have been characterized by a respiratory-dominant phenotype, often accompanied by sustained person-to-person transmission.^1^

These observations suggest that epidemic risk is not determined by virulence alone. Instead, the ability of NiV to adapt to diverse hosts and ecological contexts seems to play a pivotal role. Here, phenotypic plasticity refers to the capacity of viral populations to adjust functional behaviors under changing selective pressures and is hypothesized to represent an important facilitator of spillover risk. Conceptually, this short-term functional flexibility is distinct from quasispecies dynamics, which describe underlying genetic diversity,^2^ and from viral adaptation, which involves longer-term genetic fixation. Rather than replacing existing frameworks, this perspective complements current approaches by linking genomic signals with real-world transmission dynamics.

## Clinical heterogeneity and viral adaptation in NiV infection

The diversity of clinical presentations provides an opportunity to examine viral adaptation in real-world settings. In the Malaysian lineage (NiV-M), intensive farming conditions might have favored viral variants adapted to porcine physiology, primarily leading to encephalitic disease in humans. In contrast, the Bangladesh lineage (NiV-B) circumvents intermediate hosts, transmitting directly from *Pteropus* bats to humans via contaminated date palm sap, where it exhibits a stronger predilection for the respiratory tract.

**Figure 1:**
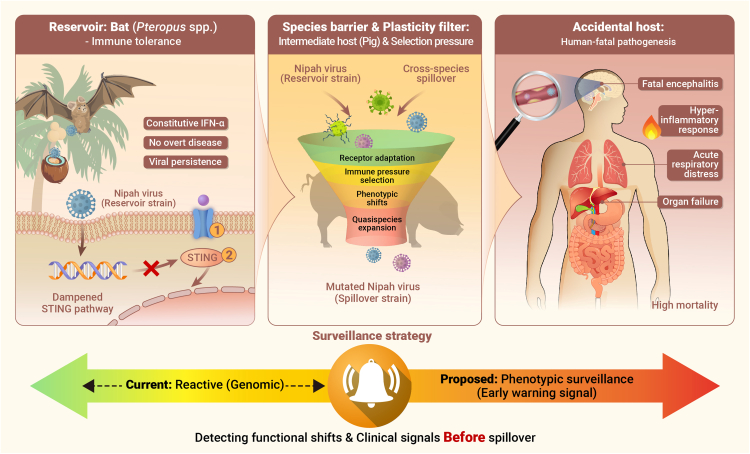
Phenotypic plasticity driving NiV spillover and the proposed surveillance shift Top: the spillover continuum. In bat reservoirs (left), NiV persists under immune tolerance. At the interspecies interface (center), a “plasticity filter” selects for variants with enhanced infectivity or immune evasion, which then cause severe hyperinflammatory disease in accidental hosts (right) due to immune mismatch. Bottom: Surveillance strategy. A proposed shift from retrospective genomic monitoring to proactive phenotypic sentinel monitoring, aiming to detect functional viral changes as early warning signals before widespread transmission.

Importantly, the observed clinical heterogeneity among outbreaks cannot be attributed exclusively to viral phenotypic shifts. These differences are profoundly confounded by ecological and host factors, particularly the route of exposure and infectious dose. For instance, the neurotropic dominance in the Malaysian outbreak may be heavily influenced by close-contact exposure to aerosolized excretions from infected pigs in intensive farming settings.^1^ Conversely, the ingestion of heavily contaminated raw date palm sap in Bangladesh presents a fundamentally different route of viral entry.^1^ In this context, phenotypic plasticity may act alongside, rather than override, the effects of ecological and exposure-related determinants (Figure 1).

## The mechanics of plasticity: Quasispecies and experimental evidence

NiV circulates within hosts as a quasispecies—a dynamic population of related variants.^2^ When the virus enters a new host, selective pressures shift rapidly, allowing variants with even small functional advantages to expand. Notably, functional phenotypic changes may occur before specific mutations become fixed. Mechanistically, this plasticity is likely mediated by conformational flexibility in viral glycoproteins, allowing NiV to tolerate minor receptor variations across hosts without immediate genetic mutation.

While direct epidemiological evidence linking plasticity to specific spillover events remains challenging to obtain, *in vitro* and *in vivo* studies provide indirect support. For instance, recent comparative models demonstrating distinct replication kinetics and dose-dependent disease progression between the Malaysia and Bangladesh isolates across varying exposure routes highlight the virus’s inherent functional flexibility.^3^ These observations should be interpreted with caution and viewed as suggestive rather than conclusive. Nevertheless, they support the possibility that clinically relevant shifts, such as increased respiratory replication, may precede detectable genomic signatures.

Importantly, the role of phenotypic plasticity in facilitating spillover and disease spread is not unique to NiV but has been increasingly recognized across other viral and ecological systems. For instance, recent modeling studies have shown that environmentally driven phenotypic plasticity in the mosquito vector (*Aedes albopictus*) can modulate the transmission dynamics and spatial spread of dengue virus.^4^ Taken together, these cross-system observations suggest that phenotypic plasticity, at both the viral and vector-host levels, may represent a broader yet underappreciated factor influencing zoonotic emergence.

## Ecological drivers and surveillance implications

NiV evolution is strongly shaped by human-driven environmental change. Deforestation and agricultural expansion increase contact between bats, livestock, and humans, creating conditions that facilitate viral adaptation and phenotypic shifts across species barriers.^5^

Currently, surveillance strategies predominantly rely on genomic sequencing, which is essential but often lags behind functional change. To better anticipate spillover risk, surveillance systems should incorporate phenotypic monitoring. Routine sequencing should be paired with rapid phenotypic assays, such as evaluating receptor affinity, at high-risk wildlife-human interfaces. Tracking clinical shifts like neurological signs in livestock or respiratory clusters in workers provides earlier warnings. In parallel, ecological indicators, like abnormal stress in bat populations,^5^ may reflect changes in viral shedding dynamics.

## Conclusion

The case of NiV highlights that the risk posed by emerging pathogens cannot be assessed solely by their lethality. Attention must also be paid to their capacity for rapid phenotypic change under ecological pressure. While directly linking phenotypic plasticity to specific spillover events remains methodologically challenging, incorporating this framework into surveillance may help shift outbreak preparedness from reactive response to earlier risk recognition, offering valuable time to intervene before localized spillover events develop into wider outbreaks. Ultimately, a One Health approach bridging molecular virology and landscape ecology is essential to transform pandemic preparedness into a proactive, predictive science.

## Funding and acknowledgments

This research project is funded in part by the Qinghai Provincial Science and Technology Program Project-Qinghai Provincial Clinical Medical Research Center for Children's Health and Diseases (2025-SF-L03) and 10.13039/501100004386Universiti Malaya private funding under project code IF038-2025.

## Author contributions

All authors contributed to the manuscript and approved the final version.

## Declaration of interests

The authors declare no competing interests.
